# Using cardiovascular risk indices to predict mortality in Covid-19 patients with acute respiratory distress syndrome: a cross sectional study

**DOI:** 10.1038/s41598-023-38732-3

**Published:** 2023-07-15

**Authors:** Martin Rief, Michael Eichinger, David West, Christoph Klivinyi, Helmar Bornemann-Cimenti, Paul Zajic

**Affiliations:** Division of Anaesthesiology and Intensive Care Medicine, Medical University of Graz, Auenbruggerplatz 5, 8036 Graz, Austria

**Keywords:** Risk factors, Comorbidities, Prognosis

## Abstract

Covid-19 patients who require admission to an intensive care unit (ICU) have a higher risk of mortality. Several risk factors for severe Covid-19 infection have been identified, including cardiovascular risk factors. Therefore, the aim was to investigate the association between cardiovascular (CV) risk and major adverse cardiovascular events (MACE) and mortality of Covid-19 ARDS patients admitted to an ICU. A prospective cross-sectional study was conducted in a university hospital in Graz, Austria. Covid-19 patients who were admitted to an ICU with a paO2/fiO2 ratio < 300 were included in this study. Standard lipid profile was measured at ICU admission to determine CV risk. 31 patients with a mean age of 68 years were recruited, CV risk was stratified using Framingham-, Procam- and Charlson Comorbidity Index (CCI) score. A total of 10 (32.3%) patients died within 30 days, 8 patients (25.8%) suffered from MACE during ICU stay. CV risk represented by Framingham-, Procam- or CCI score was not associated with higher rates of MACE. Nevertheless, higher CV risk represented by Procam score was significantly associated with 30- day mortality (13.1 vs. 6.8, *p* = 0.034). These findings suggest that the Procam score might be useful to estimate the prognosis of Covid-19 ARDS patients.

## Introduction

The Covid-19 pandemic has significantly affected individuals worldwide, causing widespread morbidity and mortality^1^. One of the most severe complications of Covid-19 is acute respiratory distress syndrome (ARDS), which has been identified as a leading cause of death among infected patients^2,3^.

In addition to respiratory complications, Covid-19 has been associated with an increased risk of cardiovascular events such as heart attacks, strokes, and thrombosis^4^. Patients with pre-existing cardiovascular disease or risk factors such as hypertension, diabetes, and obesity may be particularly susceptible to these complications^5–8^. Although the interaction between Covid-19 and cardiovascular disease is not yet fully understand, the Covid-19 pandemic has highlighted the significant impact that viral infections can have on the cardiovascular system. Patients with pre-existing cardiovascular disease, as well as those with risk factors such as hypertension, diabetes, and obesity, are at increased risk of severe illness and death from Covid-19^5^. The virus is capable of directly damaging the heart and blood vessels and triggering a systemic inflammatory response that can exacerbate existing cardiovascular conditions^7^.

There is great interest in appropriate risk assessment in Covid-19 patients, and attempts have already been made to use new scores to estimate the outcome of Covid-19 patients^9^. Cardiovascular risk scores, such as the Procam- score and Framingham score, are based on a combination of factors that have been shown to predict the risk of cardiovascular events.

Therefore, cardiovascular risk scores could be useful in predicting the risk of poor outcomes in Covid-19 patients, especially in those with preexisting risk factors. By identifying high-risk patients early, healthcare providers can implement appropriate interventions and close monitoring to prevent complications and improve patient outcomes. Possible tools for cardiovascular risk prediction could be the Framingham-, Procam- and the Charlson Comorbidity Index (CCI) score^10–14^.

To calculate these risk scores, in addition to information on pre-existing conditions current lipid parameters must be known. For the Framingham score, total cholesterol (TC) and high density lipoprotein cholesterol (HDL-C) are required, for the Procam score, low- and high density lipoprotein cholesterol (LDL-C and HDL-C) and triglycerides (TG) are mandatory^10,11^. In Covid-19 patients, several studies have been presented where the composition of standard lipids played an important role^15^. Particularly, the role of hypertriglyceridemia as a predictor for severity of Covid-19 progression should be noted^16^.

We tested the hypothesis that cardiovascular risk stratification with scoring tools such as Procam- and Framingham score or CCI may identify the increase of major adverse cardiovascular events or death during ICU stay in Covid-19 ARDS patients.

## Methods

### Study design and patient population

This study was a single-center, prospective cross-sectional study conducted at the Medical University of Graz, Austria, in a general intensive care unit. The intensive care unit, whose attending physicians were specialists in anaesthesiology and critical care medicine, primarily provided care to surgical patients. However, during the Covid-19 pandemic, the unit also provided care exclusively to patients with Covid-19. The study included all patients admitted to our Covid-19 ICU with laboratory-confirmed Covid-19 infection and a paO_2_/fiO_2_ ratio < 300, between 7^th^ of April 2020 and 31^st^ of January 2021.

### Ethical approval and informed consent

Ethics approval was obtained from the local ethics committee of the Medical University of Graz (IRB00002556) on April 7^th^, 2020, and the study was registered at clinicaltrials.gov (NCT04349982). Informed consent was obtained from all participants and/or their legal guardians, and patients who were not able to communicate due to mechanical ventilation consented to the study as soon as it was possible. Patients were free to withdraw their consent for use of their data at any given time-point, without specification of reason in accordance with Austrian and European legislation. The study has been performed in accordance with the relevant guidelines/regulations and was reported in accordance with the current STROBE guideline^17^. The study has been performed in accordance with the Declaration of Helsinki.

### Inclusion criteria

The inclusion criteria were male and female patients aged 18 years or above, with laboratory-chemically proven Covid-19 infection and a paO_2_/fiO_2_ ratio < 300 when admitted to the intensive care unit. Criteria for exclusion was defined as patient refusal. Our initial plan was to include 30 study patients for analysis, but we decided to stop the inclusion as we reached 31 patients.

### Measurements and classification at the ICU

Lipoprotein measurements were taken through a one-time extended lipid profile in addition to the admission laboratory to determine standard lipids, including triglycerides, total high and low-density lipoprotein cholesterol, which were determined by enzymatic essays as a standard procedure in the laboratory of the Medical University of Graz. Major adverse cardiovascular events were defined as stroke, myocardial infarction, pulmonary embolism, cardiac death or malignant arrhythmia (i.e., atrioventricular block III°). Patients were followed up due to estimation of mortality for a period of 90 days after ICU admission.

### Statistical analysis

Patient characteristics were described with mean and standard deviation (SD) or as numbers and percentages (%), as appropriate. Differences between measurements were analysed by t-test and Spearman correlation coefficient. The effect size was quanitified by Cohen´s d, with small effects defined within a range of 0.2 and 0.4, an intermediate effect between 0.4 and 0.8, and a large effect above 0.8. The data were analysed using SPSS v 27.0 (IBM Corp. Released 2020. IBM SPSS Statistics for Windows, Version 27.0. Armonk, NY: IBM Corp).

## Results

This study presents the results of 31 patients admitted to the intensive care unit (ICU) with severe Covid-19. The mean age of the patients was 68 years (with a range of 59–79 years), and 22 were male while 9 were female. The mean body mass index was 28 kg/m^2^ (with a range of 22–34 kg/m^2^) (Table 1).

**Table 1:** Baseline characteristics.

Baseline characteristics	N = 31
Age	68 [59;79]
Gender (male/female)	22M/9F
Body mass index (kg/m^2^)	28 [22;34]
Previous illness	
Diabetes	12 (38.7)
Arterial hypertension	25 (80.6)
Cardiovascular disease	18 (58.1)
Cerebrovascular disease	3 (9.7)
Chronic pulmonary disease	6 (19,4)
Malignancy	6 (19.4)
Transplantation	3 (9.7)
Smoking	9 (29.0)
Operation (within 7 days prior ICU)	10 (32.3)
Statin therapy	10 (32.3)

Displayed as median with interquartile range in brackets or percentages in round brackets. M = male. N = number. F = female.

The baseline characteristics of the patients showed that 12 of them had diabetes (38.7%), 25 had arterial hypertension (80.6%), 18 had cardiovascular disease (58.1%), 3 had cerebrovascular disease (9.7%), and 6 had malignancy (19.4%) (Table 1). Additionally, 3 patients had undergone transplantation (9.7%), 9 were smokers (29%), and 10 had undergone an operation within 7 days prior to ICU admission (32.3%) (Table 1). 10 patients were on statin therapy (32.3%).

The ICU characteristics (Table S1) showed that the mean hospital stay before ICU admission was 7.35 days (with a range of 10.8 days), and the mean length of stay in the ICU was 14 days (with a range of 12.6 days). The 30-day mortality rate was 32.3%, while the 90-day mortality rate was 9.7%. Nine patients underwent tracheostomy (29%), and 5 suffered from pneumothorax (16.1%). Eight patients suffered from major adverse cardiovascular events as stroke, myocardial infarction or cardiac death (25.8%) (Table S1).

Standard lipid levels displayed in Table 2, with triglycerides already at the mean very close to the upper limit of 150 mg/dl (148.7 mg/dl with a standard deviation of 50.6 mg/dl). Triglycerides were significantly associated with 30-day mortality (*p* = 0.035). Total-, low- and high-density lipoprotein cholesterol showed no significant associations with 30-day mortality (Table 2).

**Table 2:** Standard lipids and association with 30-day mortality.

Standard lipids	Mean	SD	Death	SD	Survivor	SD	p*
LDL-C	73.6	34.4	80.8	28.9	70.1	36.9	0.427
HDL-C	29.4	10.0	28.9	8.2	29.7	11.0	0.846
TC	137.3	40.8	147.6	32.4	132.4	44.2	0.342
TGL	148.7	50.6	176.1	54.9	135.6	43.9	0.035**

Displayed as means in mg/dl, standard deviations and correlation coefficients with significance p value. * calculated with t-test for non depending variables. **significance level *p* < 0.05 HDL-C = high density lipoprotein cholesterol. LDL-C = low density lipoprotein cholesterol. mg/dl = milligram per deciliter. SD = standard deviation. TC = total cholesterol. TGL = Triglycerides.

Table 3 presents the mean scores, death and survivor rates, and statistical significance for the Procam-, Framingham-, and Charlson Comorbidity Index (CCI) scores in relation to 30-day mortality in the studied population.

**Table 3:** Risk scores and association with mortality.

Score	Mean (SD)*	Death*	Survival*	*p*^*§*^
*Procam*	8.6 (8.3)	13.1 (9.6)	6.5 (6.8)	0.034**
*Framingham*	13.4 (8.9)	13.4 (7.3)	13.4 (9.7)	0.996
*CCI*	42.5 (36.4)	32.6 (30.7)	47.2 (38.7)	0.306

^§^calculated with t-test for non depending variables. * values in percent. ** significance level < 0.05. CCI = Charlson Comorbidity Index. *p* = p value. SD = standard deviation.

The Procam score had a mean of 8.6 (SD 8.3) and showed a significant association with 30-day mortality (*p* = 0.034), with a higher score indicating a higher mortality rate. The Framingham score had a mean of 13.4 (SD 8.9) but did not show a significant association with 30-day mortality (*p* = 0.996). The CCI had a mean of 42.5 (SD 36.4) and did not show a significant association with 30-day mortality (*p* = 0.306).

When analyzing the effect size, the Procam score had a large effect on the 30-day mortality (Cohen d = 0.849), while the other two parameters did not reach a significant effect.

The correlations between the risk scores and mortality or the occurrence of MACE were shown graphically in Supplemental Figure S1 and Figure S2. In Figure S1, the weighting with more deaths is evident at higher Procam scores, in contrast to Framingham and CCI scores, where a weighting in the direction of survivors can be seen at higher score values. In Figure S2, no correlations between the risk scores and the occurrence of MACE are apparent.

Furthermore, the correlations between standard lipoproteins on admission to the ICU and 30-day mortality or the occurrence of MACE are shown in Supplemental Figure S3 and Figure S4. Figure S3 shows a weighting of higher triglyceride values in patients who died in the intensive care unit, whereas this cannot be shown for the other standard lipoproteins. In Figure S4 no higher standard lipoprotein values could be shown with the occurrence of MACE.

We further analyzed the positive predictive value (PPV) and negative predictive value (NPV) of the Procam score for prediction of death within the first 30 days after ICU admission. We thought that a Procam score of 8.6 (as the mean value in our population) would be the ideal value, that resulted in a PPV of 0.55 and a NPV of 0.8. The sensitivity was 0.6 and specificity 0.76.

## Discussion

This study demonstrates the significant association between cardiovascular risk represented by risk scores and mortality in patients with severe Covid-19 and ARDS. The three different risk scores investigated in this study were derived from different patient collectives and also require different parameters to calculate them. The Framingham risk score is a well-established tool for predicting cardiovascular risk in individuals based on various risk factors such as age, sex, cholesterol levels, blood pressure, and smoking status. Primarily, the score was used to predict the risk of myocardial infarction within the next 10 years.^10^ The Procam (Prospective Cardiovascular Munster) score is a risk assessment tool that was developed to identify individuals at high risk of developing cardiovascular disease, respectively, the risk of suffering a heart attack within 10 years^11^. The score considers several risk factors, including age, gender, smoking status, blood pressure, cholesterol levels, and family history of cardiovascular disease^11^.

Currently, there are already several scientific studies on the association of different scores on the severity or mortality of Covid-19 patients^12,13^, such as the Charlson Comorbidity Index (CCI), which is said to have the best predictive capability for severe outcome of hospitalized Covid-19 patients compared to other scores^14^.

The CCI is a valuable tool for estimation of the survival chances of patients who suffer from multiple health problems and calculates the percentage probability of 10-year survival^14^. This index is based on 17 different health conditions that have been associated with mortality rates, such as heart disease, cancer, diabetes, liver disease, and others. The index assigns scores to each condition based on its severity, with higher scores indicating more severe conditions. The CCI has been used to predict short- and long-term outcomes for patients, including their ability to function, the length of their hospitalization, and their mortality rates. In the context of Covid-19, the CCI can help clinicians estimate the prognosis for patients who are at higher risk of developing severe illness due to underlying health conditions. For instance, patients with heart disease, diabetes, and other chronic conditions have been shown to be more vulnerable to severe Covid-19 infections, and the CCI can help predict their outcomes^14^.

To date, it has not been investigated whether any of these three different risk scores have any significance in Covid-19 ARDS patients.

Our results demonstrate that the Procam score a tool for measuring cardiovascular risk, significantly predicts mortality in critically ill Covid-19 ARDS patients, while the Framingham Risk Score and the CCI did not differ significantly. This may be due to the fact that the Procam score considers triglycerides, low- and high density lipoprotein cholesterol values. Framingham score considers only high density lipoprotein cholesterol and total cholesterol values and the CCI does not consider lipid values. Some studies have suggested that low density lipoprotein cholesterol levels may be associated with a higher risk of severe Covid-19, while high triglyceride levels may be associated with worse outcomes^16,18^. It has already been shown in many studies in animals and humans that triglyceride levels increase as a result of various cytokines or their release^19^. Accordingly, it is reasonable to assume that this also applies to Covid-19 ARDS patients and that higher levels of triglycerides are subsequently associated with increased mortality, as shown in our study.

Furthermore, we did not find any association of cardiovascular risk and the occurrence of MACE during the ICU stay in Covid-19 patients. We could not find any reason for this, possibly bacterial superinfection as already suggested by Musuuza et al. is the predominant cause of the death of Covid-19 patients and maybe influences the results through a very rapid lethal course^20^.

One potential mechanism underlying the association between cardiovascular risk represented by the Procam score and mortality in Covid-19 ARDS patients is direct damage to the heart and blood vessels by the virus. SARS-CoV-2 has been shown to infect endothelial cells and cause endotheliitis, which can lead to thrombosis and other cardiovascular complications^21^. Furthermore, the systemic inflammatory response triggered by the virus can also contribute to cardiovascular complications and worsen existing conditions.

The Procam score has been previously validated as a tool for predicting cardiovascular risk in the general population^11^. Our study demonstrates its utility in predicting mortality in Covid-19 ARDS patients, highlighting the importance of identifying and managing cardiovascular risk factors in these patients.

Our calculated PPV yields a PPV rate of 55% and an NPV of 80% are promising results which encourage further evaluation of this risk score as prognostic parameters for mortality in an ICU-setting. However, it has to be kept in mind that our sample represents a highly specific cohort. Therefore, generalizations should only be made cautiously. Foremost, the score and any cut-offs must be validated in an independent sample.

### Limitations

While our study on the association between cardiovascular risk and mortality in Covid-19 ARDS patients provides important insights into this high-risk population, it is important to acknowledge several limitations that may impact the generalizability of our findings.

One significant limitation of our study is the low number of patients included for this research question. While we had a sufficient sample size to detect significant associations between Procam score, triglycerides, and mortality, a larger sample size may have provided more robust results and allowed for more in-depth analysis of the relationships between these variables. By analysing the effect size, we could, however, demonstrate that the observed effect is large, which also puts the group size into perspective.

Another limitation of our study is that we only measured lipid levels at the admittance to the intensive care unit. This means that we do not have information on how lipid levels may have changed over time, and we cannot determine whether changes in lipid levels during ICU stay may have impacted outcomes. Future studies may benefit from measuring lipid levels at multiple time points throughout hospitalization to better understand the relationship between lipid levels and outcomes in Covid-19 ARDS patients.

Finally, our study was conducted at a single center, which may limit the generalizability of our findings to other populations and settings. Additional research is needed to determine whether similar associations between Procam score, triglycerides, and mortality are observed in other patient populations and healthcare systems.

## Conclusion

In conclusion, our study provides important insights into the association between cardiovascular risk factors and mortality in Covid-19 ARDS patients admitted to the intensive care unit. Our findings highlight the importance of identifying and managing pre-existing cardiovascular disease and risk factors in these patients. The Procam score remains as an important tool for assessing cardiovascular risk and may be an important factor for predicting mortality in Covid-19 ARDS patients. Further research is needed to fully understand the mechanisms underlying these associations and to develop more targeted and effective strategies for managing cardiovascular risk in this high-risk population. Ultimately, a comprehensive approach to managing Covid-19 ARDS patients that takes into account both respiratory and cardiovascular factors may be essential for improving outcomes and reducing mortality.

## Supplementary Information

Supplementary Information.

## Data Availability

The dataset used during the current study is available from the corresponding author on reasonable request.
