# Supplementary material for: Using cardiovascular risk indices to predict mortality in Covid-19 patients with acute respiratory distress syndrome: a cross sectional study
Source: Sci Rep. 2023 Jul 15;13:11452. doi: 10.1038/s41598-023-38732-3 (PMC10349805; doi:10.1038/s41598-023-38732-3)
Supplement: Supplementary file 1 — Supplementary Information. [file 41598_2023_38732_MOESM1_ESM.docx]

SUPPLEMENTAL MATERIALS

Using Cardiovascular Risk Indices to predict Mortality in Covid-19 Patients with Acute Respiratory Distress Syndrome – A Cross Sectional Study

Martin Rief, MD PhD^1^

Michael Eichinger, MD MSc^1^

David West, MD^1^

Christoph Klivinyi, MD^1^

Helmar Bornemann-Cimenti, MD MSc PhD^1*^

Paul Zajic, MD PhD^1^

^1^ Division of Anaesthesiology and Intensive Care Medicine, Medical University of Graz, Austria

*corresponding author

**Figure S 1**: Risk scores and 30 day mortality.


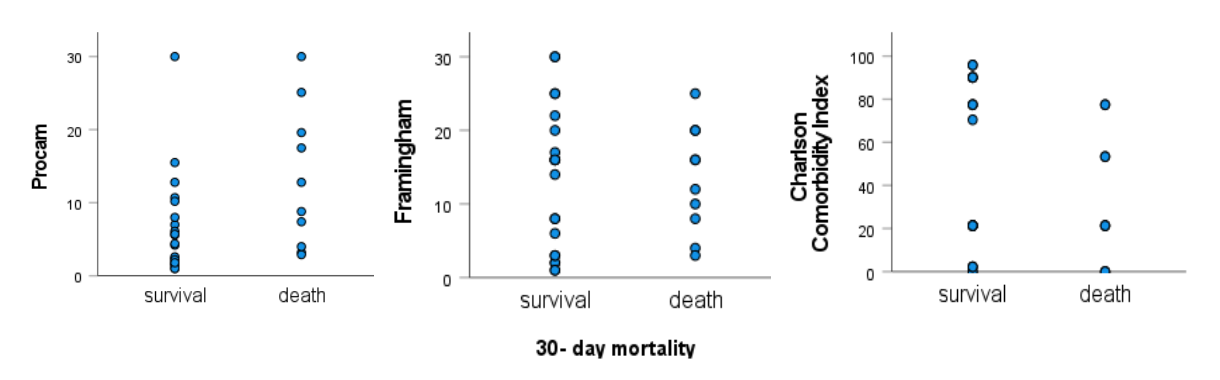


**Legend:** X axis with number of patients and y axis with percentage of the respective score at intensive care unit (ICU) admission. Number of patients with survival shown with blue bars on the left side. Number of patients died during ICU shown on the right side with red bars.

**Figure S 2**: Risk scores and major adverse cardiovascular events.


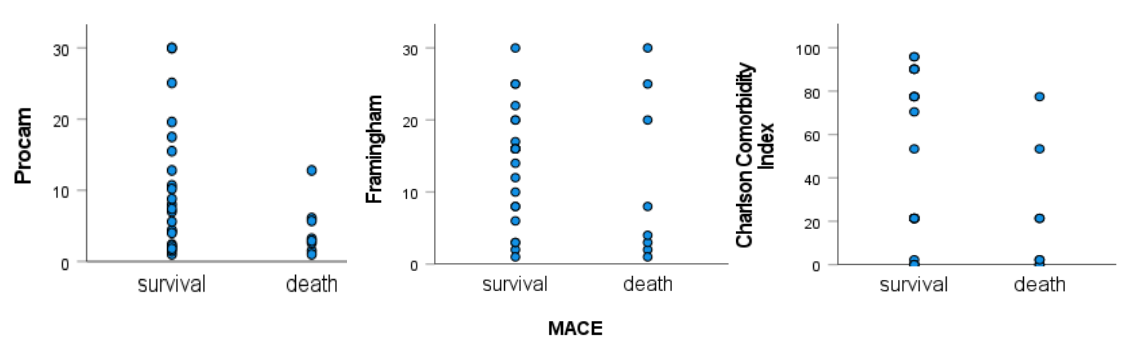


**Legend:** MACE= major adverse cardiovascular events. X axis with number of patients and y axis with percentage of the respective score at intensive care unit (ICU) admission. Number of patients with no MACE during ICU shown with blue bars on the left side. Number of patients with MACE during ICU shown on the right side with red bars.

**Figure S 3**: Standard lipoproteins and 30 day mortality.


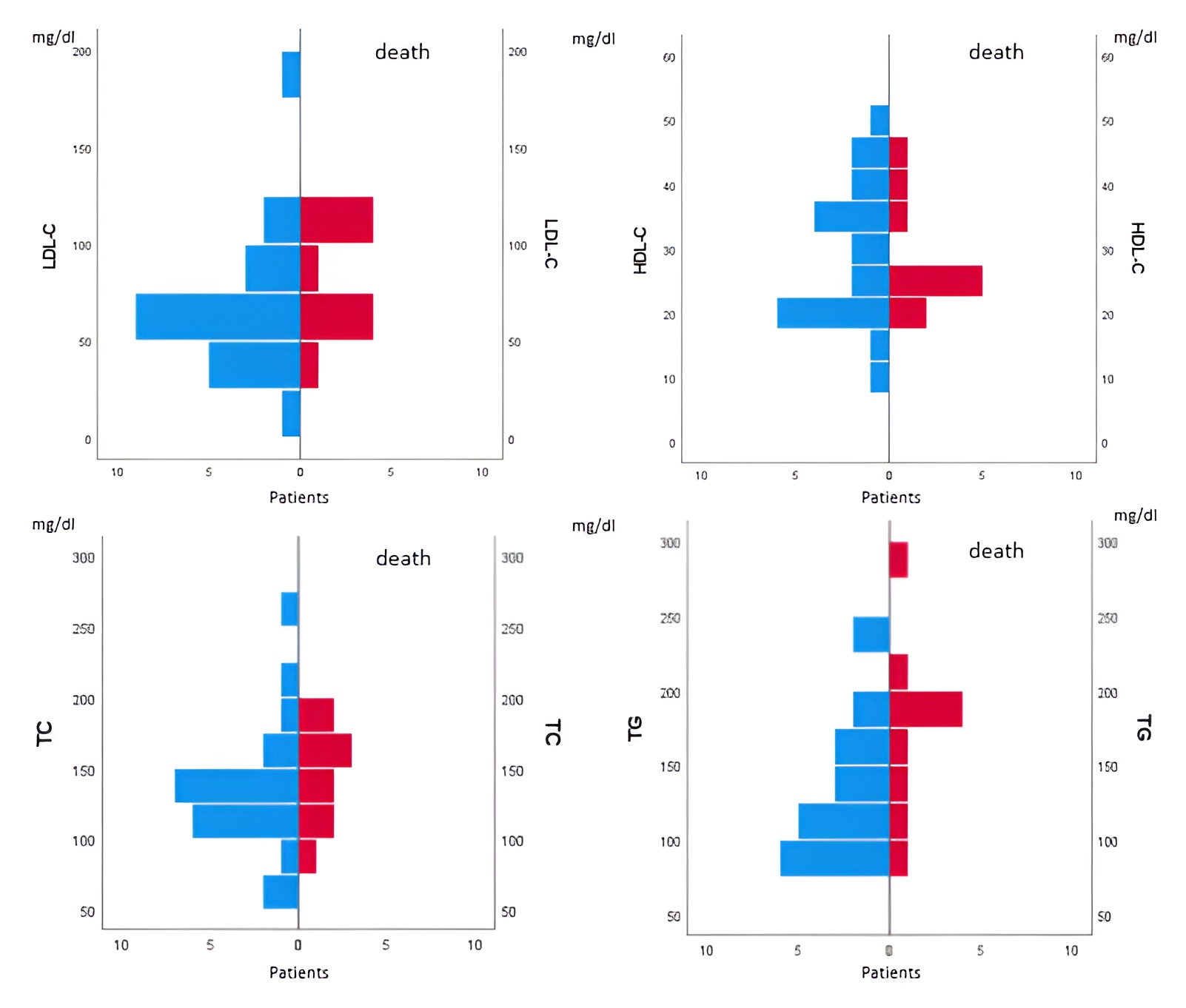


**Legend:** X axis with number of patients and y axis with values of the respective lipoprotein parameters at intensive care unit (ICU) admission. Number of patients with survival shown with blue bars on the left side. Number of patients died during ICU shown on the right side with red bars.

**Figure S 4**: Standard lipoproteins and major adverse cardiovascular events.


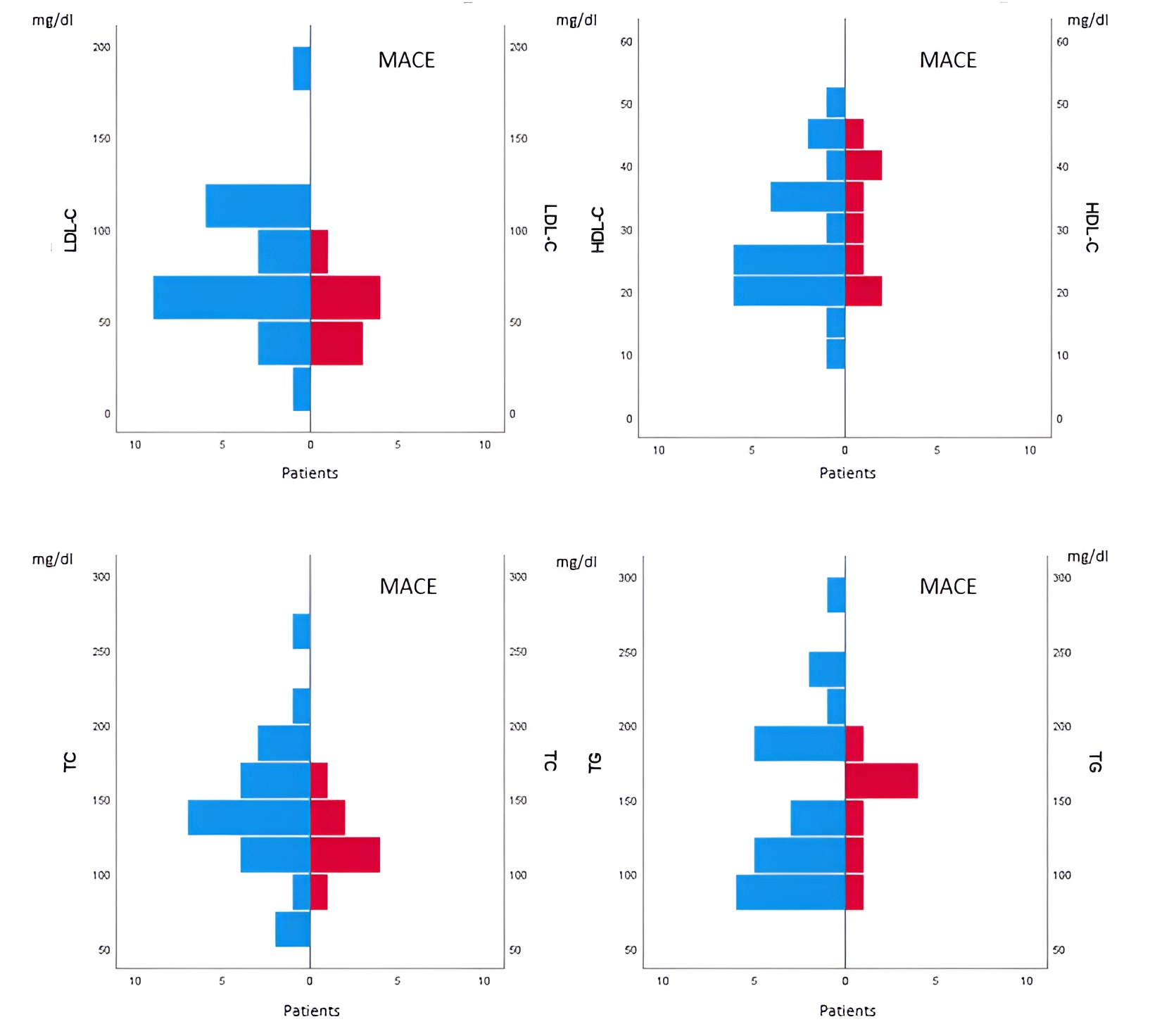


**Legend:** MACE= major adverse cardiovascular events. X axis with number of patients and y axis with values of the respective lipoprotein parameters at intensive care unit (ICU) admission. Number of patients with no MACE during ICU shown with blue bars on the left side. Number of patients with MACE during ICU shown on the right side with red bars.

**Table S 1:** Intensive care unit characteristics.

| **ICU characteristics** | **N=31** |  |
| --- | --- | --- |
| Hospital stay before ICU*  Length of stay at ICU* | 7.35 [10.8]  14 [12.6] |  |
| 30 day mortality | 10 (32.3) |  |
| 90 day mortality  Tracheostomy** | 3 (9.7)  9 (29.0) |  |
| Pneumothorax** | 5 (16.1) |  |
| MACE**  Discharged home | 8 (25.8)  19 (61.3) |  |

**Legend:** Displayed as mean value with standard deviation in angular brackets and numbers with percentages in round brackets. * in days. **during ICU. ICU= intensive care unit. MACE= major adverse cardiac events. N= number.
